# c-index and Subindices of the h-index: New Variants of the h-index to Account for Variations in Author Contribution

**DOI:** 10.7759/cureus.2629

**Published:** 2018-05-15

**Authors:** Alex Post, Adam Y Li, Jennifer B Dai, Akbar Y Maniya, Syed Haider, Stanislaw Sobotka, Tanvir F Choudhri

**Affiliations:** 1 Neurosurgery, Mount Sinai School of Medicine; 2 Neurosurgery, The Icahn School of Medicine at Mount Sinai, New York, USA; 3 Surgery, Montefiore Medical Center, New York, USA

**Keywords:** hp-index, hi-index, hs-index, cp-index, cs-index, co-index, bibliometrics, scienometrics, academic neurosurgery

## Abstract

Objectives

Bibliometrics are used to assess or compare the academic productivity of individuals or groups. Most of these metrics, including the widely used *h*-index, do not recognize the added contribution that is generally provided by authors listed first, second, second-to-last and last (enhanced positions) in a publication citation. We propose the *c*-index as a novel modification to the *h*-index that will better reflect an individual’s academic output, incorporating authorship position.

Methods

One hundred and sixty-six academic neurosurgeons in eight New York City (NYC) metropolitan region training programs were identified through department websites. Using the Scopus citation database, bibliometric profiles were created for each surgeon. Once an individual’s *h*-index was calculated, the *h*-core articles (those with *h* or more citations) were specifically assessed to determine citation author position. Novel bibliometric indices were created to reflect the number of *h*-core articles that accounted for primary (*h*_p_), senior (*h*_s_) or internal authorship (*h*_i_) position. Weighted “involvement factors” for primary (*i*_p_) and senior (*i*_s_) author contribution were created to reflect the added value of “enhanced position” authorship in an individual’s *h*-core publications. *c*-indices were created to reflect the author’s *h*-index once augmented by primary (*c*_p_), senior (*c*_s_), and overall (*c*_o_) “enhanced position” authorship. Comparisons were made within each institution and across institutions, according to academic rank (assistant professor, associate professor, professor and chairperson).

Results

Breakdown by academic rank showed an increasing average *h*-index progressing from assistant professor through professor rank with no significant difference demonstrated between professor and chair status. This pattern was seen across all departments (aggregate) but with fewer instances of significance at the level of individual departments. After *h*-index modification, *c*_p_, *c*_s_, and *c*_o _indices showed a similarly significant trend. As faculty rank increased, there was a significant trend toward increasing numbers of articles with authors in enhanced positions and a higher percentage of articles with the author in a senior position. Academic faculty had higher *h*, *c*_p_, and *c*_s _indices than clinical faculty. Evaluation of each individual department revealed no significant trend regarding a department’s higher average *c*_p_ or *c*_s_. Average *c*-index for a department paralleled the average *h*-index of that department, with larger departments tending to have larger cumulative *h*, *c*_p_, *c*_s,_ and *c*_o_ indices. No consistent correlation was seen between mean *h*-indices and academic rank at an individual departmental level.

Conclusions

This study examines the academic productivity of a subset of neurosurgical programs in the NYC metropolitan area as a test bed for novel bibliometric indices. *h*_p_, *h*_i_, and *h*_s_ represent the respective number of primary, internal and senior authorship papers that comprise an individual’s *h*-core papers. *c*_p_, *c*_s_, and *c*_o_, variations of the *h*-index metric, are designed to more accurately reflect the contributions by primary, secondary and senior authors. Increasing academic rank was associated with an increased number of articles with the author in enhanced positions and a higher percentage of articles in a senior position.

## Introduction

The academic output of an individual medical professional or department may be measured in many different ways, each having its own subjective value to the evaluator. Previously used metrics include quantity and type (abstract, journal article, chapter) of publications, quality of research (level of evidence, prestige or impact factor of the journal of publication) and associated funding (grants awarded by the National Institutes of Health, for-profit or non-profit private foundations). Bibliometrics attempts to quantify consistent comparisons between or among individuals or groups. The results may be used for individual self-evaluation over time, individual evaluation for academic promotion, and for comparison among members within or between departments.

The most used citation metric is the *h*-index, described by Jorge Hirsch in 2005 [1]. Defined as the number of papers (*h*) with at least *h* citations, this metric attempts to balance the quantity of an author’s publications with an assessment of each article’s quality and relevance to the literature. Rousseau later proposed the Hirsch core (*h*-core) as “the set consisting of the first *h* articles” or only those articles with *h* or more citations [2]. Drawbacks to the *h*-index include possible influence of self-citation, inclusion of articles that are speculative, widely criticized or noteworthy for conclusions that are later refuted, and failure of the metric to appreciate highly influential articles cited well in excess of *h* citations. The *h*-index is also time-dependent; articles often require time to be fully appreciated before they are cited in other works, which tends to benefit older articles and researchers who have had more time for their *h*-index to accrue.

To compensate for the drawbacks of the *h*-index, many variations or adaptations have been created. Hirsch’s *m*-quotient [1] attempted to eliminate *h*-index bias toward older articles and researchers. The *g*-index [3] and *e*-index [4] attempt to rectify bias against authors with heavily cited articles that greatly exceed the *h*-index. A 2011 article by Bornmann et al. [5] evaluated 37 different variants of the *h*-index, noting a mean correlation coefficient of the *h*-index to its variants from 0.8 to 0.9 and a relative redundancy between the *h*-index and most of its variants. Whether the *h*-index or one of its variants are used, each of these metrics relies on article citation with equal weight given to each author, regardless of his or her position in a publication’s author list.

Any multi-author publication contains inherent differences in the amount and type of contribution each author contributes to the final product. This collaboration is represented, in part, by each author’s respective position in the publication title with the assumption of increased involvement of the first and last authors, slightly lesser input from the second and second-to-last authors, and so on as authors take more internal positions in the citation list. Some prior papers have sought to evaluate the relative authorship position or value [6] or to modify the relative weight given to each author. Howard [7] gave each article a total value of one point, with each author receiving a fraction so that the summation of all authors was one. Romanovsky [8] proposed a revised *h*-index that weighted first and last-author papers by 1.6 and middle author papers by 0.4. Lee et al. [9] performed calculations on a subset of individuals in their paper, giving a weight of 1.0 to first and last authors, 0.5 to the second author and 0.25 to the remaining authors.

The distillation of an individual’s academic effort into numerical format is enticing because it allows for easy comparison, but it also risks oversimplification. We propose the *c*-index as a new variant of the *h*-index, with enhanced recognition for the primary (first and second) and senior (last and second-to-last) positions in the authorship list of a multi-authored paper. This format will allow for a more detailed understanding of each author’s *h*-index and the types of academic contributions they have made to their *h*-core articles and their entire set of published works.

## Materials and methods

Selection of programs and surgeons

A listing of the 2017 neurosurgery residency training programs was obtained from the Accreditation Council for Graduate Medical Education (ACGME) [10] and programs within a 30-mile radius of New York City (NYC) were selected. These programs included Albert Einstein College of Medicine at Montefiore Medical Center, Hofstra Northwell School of Medicine, Icahn School of Medicine at Mount Sinai, New York Medical College at Westchester Medical Center, New York Presbyterian Hospital (Columbia campus), New York Presbyterian Hospital (Cornell campus), New York University School of Medicine and Rutgers New Jersey Medical School.

Department websites were evaluated to identify the chair and to create a list of faculty members and their respective academic ranks (instructor, assistant professor, associate professor and professor). Faculty members included in this study were surgeons with neurosurgery listed as their primary specialty. Adjunct faculty with a non-neurosurgical primary specialty (e.g., radiology, orthopedics, otolaryngology, rehabilitation medicine) and non-surgeons (e.g., neurologists, radiologists, physiatrists, nonsurgical Ph.D.’s) were excluded from this study. 

Definition of citation metrics

*h*-index: Hirsch’s *h*-index, for an individual, is defined as the number of papers, *h*, each having at least *h* citations. *h*_p_, *h*_s_, *h*_i_, *c*_p_, *c*_s_, and *c*_o_ were calculated using only the *h*-core articles.

*h*_p_: The number of *h*-core articles with the author in first or second authorship position. *h*_s_: The number of *h*-core articles with the author in last or second-to-last authorship position. *h*_i_: The number of *h*-core articles with the author in an internal position (not first, second, second-to-last or last position).

*i*_p_ = (# first-author *h*-core articles) + 0.5 (# second-author *h*-core articles) *i*_s_ = (# last-author *h*-core articles) + 0.5 (# second-to-last author *h*-core articles if ≥ four authors)

*c*_p _= *h* + *i*_p_                (*h*-index + augmentation due to primary author involvement) *c*_s _= *h* + *i*_s                      _(*h*-index + augmentation due to senior author involvement) *c*_o _= *h* + *i*_p_ + *i*_s_          (*h*-index + augmentation due to primary and senior author involvement)

Calculation of *h*-index

Each faculty member was located within the Scopus abstract and citation database using the “Author Search” feature [11] with the author’s last name, first and middle initials as search input strings. Author identification included evaluation of the author’s name (with variations), location, site of academic affiliation, affiliations with other authors, journals and manuscript titles, and known publications by the author. Once the author was appropriately identified, an automated calculation of *h*-index was performed via the Scopus platform. The author’s *h*-core articles were identified and were used to calculate the *h*_p_, *h*_s_, and *h*_i_ indices as per the above definitions.

Bonus points added to *h*-index based on number of authors

After automated calculation of an author’s *h-*index, the subset of articles used to generate that *h*-index was inspected manually to determine the author’s position in the authorship list of each article. *c*_p_, *c*_s_, and *c*_o_ were then calculated by adding bonus points to the *h*-index (Tables 1, 2).

**Table 1:** Additional points applied based on author position. Additional points used for calculation of *i*_p_ and *i*_s_.

# Authors	Points added to h-index
1	One point
2	One point for 1^st^ or last authorship
3	One point for 1^st^ or last authorship, 0.5 points for 2^nd^ authorship
4+	One point for 1^st^ or last authorship, 0.5 points for 2^nd^ or 2^nd^-to-last authorship

**Table 2:** Additional points applied based on author position. Additional points used for calculation of *c*_p_, *c*_s_, and* c*_o_.

#Authors	Points applied to c_p_	Points applied to c_s_	c_o_
1	One point	No	Yes
2	1^st^ position (one point)	Last position (one point)	Yes
3	1^st^ position (one point) or 2^nd^ position (0.5 pt)	Last position (one point)	Yes
4+	1^st^ position (one point) or 2^nd^ position (0.5 pt)	2^nd^-to-last (0.5 pt.) or last position (one point)	Yes

Statistical analysis

The following *a priori* comparisons were performed:

1) *h*-index, *c*_p_, *c*_s_, and *c*_o_ vs. academic rank (aggregate department)

Data for 166 academic neurosurgeons from eight neurosurgical departments was broken down by academic ranks of instructor, assistant professor, associate professor, professor and chair. Mean *h*-index for each rank was calculated, and significant differences between mean *h*-index and academic rank were tested using one-way analysis of variance (one-way ANOVA). The same procedure was used to test for differences between mean *c*_p_, *c*_s_, and *c*_o_ indices and faculty rank. Differences between indices within a particular faculty rank were tested using one-way ANOVA.

2) *h*_p_, *h*_s_, *h*_p_/*h*, *h*_s_/*h* vs. academic rank (aggregate department)

Mean *h*_p_ for each academic rank was calculated and significant differences between mean *h*_p_ and academic rank were tested using one-way ANOVA. Mean *h*_s_, *h*_p_/*h*, and *h*_s_/*h* were also calculated for each academic rank, and significant differences between mean index values and academic rank were retested using one-way ANOVA. 

3) *h*-index, *c*_p_, *c*_s_, *c*_o_ vs. faculty category vs. faculty rank (aggregate department)

Faculty members were distinguished by ranks of assistant, associate and professor, as well as clinical rank (i.e., having the words adjunct, affiliate, clinical, courtesy, voluntary, instructor, lecturer, attending, preceptor or staff in their title) and academic rank (i.e., assistant professors, associate professors, professors and chairpersons) faculty categories. Department chairs could not be compared because there were no clinical chairs in the eight programs studied. Mean *h*-index was calculated for each faculty rank within both faculty categories. Differences between faculty ranks within each faculty category, as well as differences between faculty categories, were tested for significance using two-way ANOVA. The same procedure was used to test *c*_p_, *c*_s_, and *c*_o_ indices.

4) *h*-index, *c*_p_, *c*_s_, and *c*_o_ vs. academic rank (individual department)

Mean *h*-index for each faculty rank in individual NYC neurosurgery programs was calculated, and significant differences between mean *h*-index and academic rank within each program were tested using one-way ANOVA.

5) *h*-index vs. program size

Mean *h*-index for individual NYC neurosurgery programs was plotted as a function of program size. A Pearson correlation coefficient was obtained for the data and used to test for significant linear correlation. The same procedure was used to test for correlations between mean *c*_p_, *c*_s_, and *c*_o_ indices and program size.

6) Departmental mean *h*-index vs. percentage of each academic rank

Mean *h*-index for individual NYC neurosurgery programs was plotted as a function of percentages of faculty ranks within individual NYC neurosurgery programs. A Pearson correlation coefficient was obtained for each faculty rank and used to test for significant linear correlation.

7) Proportional odds model for faculty rank as a function of *h*-index, *c*_p_, *c*_s_, *c*_o_

The proportion of times the model, an extension of multinomial logistic regression, made the correct prediction in sample of faculty rank based on *h*-index, *c*_p_, *c*_s_, and *c*_o_ indices was generated. The success rates of each index were obtained for the data and used to test for significant differences between each other using one-way ANOVA.

Data was acquired and calculations were performed from November–December 2017. All statistical analyses were calculated using GraphPad PRISM (La Jolla, CA) and RStudio (Boston, MA). Significant values were considered to be p < 0.05 and mean values are presented with standard error of the mean (SEM). Institutional Review Board (IRB) approval was not required because patient information was not analyzed in this study.

## Results

As anticipated, mean *h*-indices for NYC neurosurgeons increased as faculty rank increased. The mean *h*-indices were 8.42 for assistant professors (range (0-29), mean 8.42 (SEM 0.75)), 16.26 for associate professors (range (1-78), mean 16.26 (SEM 2.18)), 33.46 for professor (range (5-113), mean 33.46 (SEM 3.17)), and 38.63 for chairpersons (range (30-62), average = 38.63 (SEM 3.79)). With a single faculty member at the instructor level (*h*-index = 2), this category was underpowered and was not included in further comparative analysis (Table 3).

**Table 3:** Overview of mean indices for New York City (NYC) neurosurgeons.

Rank	n	Min	Max	Mean h (SEM)	Mean c_p_ (SEM)	Mean c_s_ (SEM)	Mean c_0_ (SEM)
Assistant	66	0	29	8.42 (0.75)	11.75 (1.01)	9.58 (0.90)	12.90 (1.13)
Associate	43	1	78	16.26 (2.18)	21.23 (2.65)	20.43 (2.84)	25.41 (3.28)
Professor	48	5	113	33.46 (3.17)	42.68 (3.75)	44.49 (4.54)	53.71 (5.00)
Chair	8	30	62	38.63 (3.79)	47.06 (4.29)	52.00 (5.53)	60.44 (5.87)

**Figure 1:**
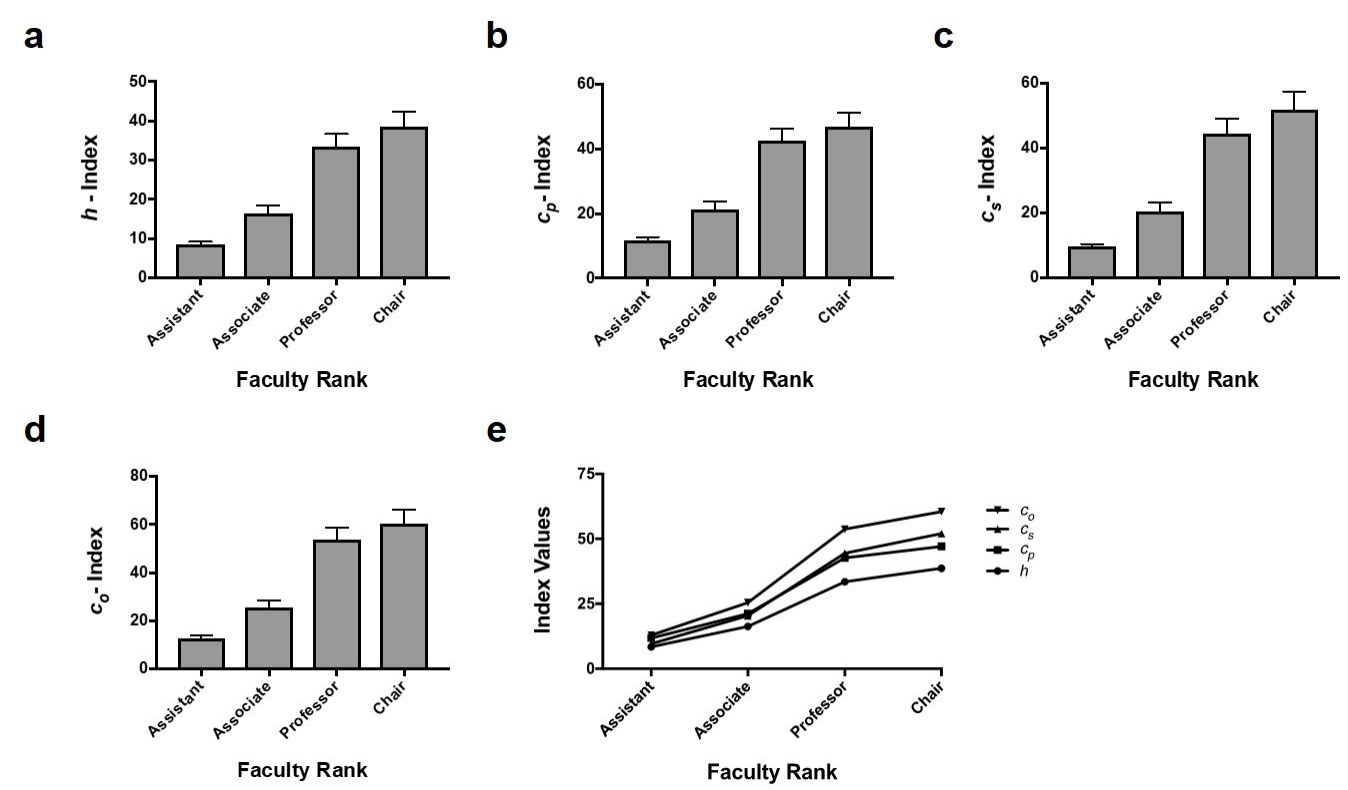
Mean indices vs. academic rank. *h*, *c*_p_, *c*_s_, and *c*_o_ indices reflect academic productivity in New York City (NYC) neurosurgery programs. (a-d) Bar graphs show mean (SEM) index values calculated from Scopus for NYC neurosurgeons with different faculty ranks. Graphs a, b, c, and d show mean *h*,* c_p_*,*c_s_*,and*c_o_*indices, respectively. (e) Line graph compares mean *h*,* c_p_*,*c_s_*,and*c_o_*indices for NYC neurosurgeons with different faculty ranks.

One-way ANOVA testing showed there was a significant difference between average *h*-index and academic rank (p < 0.0001). With the exception of chairpersons vs. professors, Tukey’s multiple comparisons test showed significant differences between all rank pairs: chairpersons vs. associate professors (p = 0.0006), chairpersons vs. assistant professors (p < 0.0001), professors vs. associate professors (p < 0.0001), professors vs. assistant professors (p < 0.0001), and associate vs. assistant professors (p = 0.0352) (Figure 1a, Table 4).

**Table 4:** Tukey’s multiple comparisons tests for faculty rank at different indices (one-way ANOVA). APV: Adjusted p-value; Assoc.: Associate professor; Asst.: Assistant professor; Prof.: Professor; Sig.: Significance.

	h-index		c_p_-index		c_s_-index		c_0_-index	
	Sig.	APV	Sig.	APV	Sig.	APV	Sig.	APV
Chair vs. Prof.	NS	0.7920	NS	0.9142	NS	0.7671	NS	0.8659
Chair vs. Assoc.	***	0.0006	**	0.0011	***	0.0005	***	0.0006
Chair vs. Asst.	****	<0.0001	****	<0.0001	****	<0.0001	****	<0.0001
Prof. vs. Assoc.	****	<0.0001	****	<0.0001	****	<0.0001	****	<0.0001
Prof. vs. Asst.	****	<0.0001	****	<0.0001	****	<0.0001	****	<0.0001
Assoc. vs. Asst.	*	0.0352	*	0.0330	*	0.0352	*	0.0286

Subsequent calculations of mean *c*_p_, *c*_s_, and *c*_o_ indices revealed similar findings. Each index showed no statistically significant difference between professor and chair rank, but did show statistically significant differences between all other rank pairs (Figure 1b-1d, Table 4).

Comparisons of indices within particular faculty ranks using one-way ANOVA tests showed statistical significance of *h*-index vs. *c*_o_ at the chair (p = 0.0206), professor (p = 0.0046) and assistant professor (p = 0.0063) levels.

**Figure 2:**
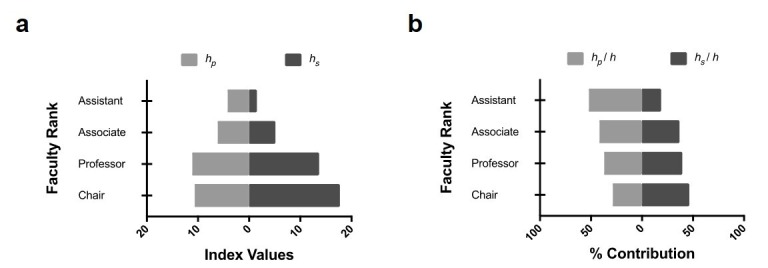
Primary and senior authorship as a function of faculty rank. (a) Tornado chart shows mean index values for a subset of *h*-core articles for New York City (NYC) neurosurgeons with different faculty ranks. Articles accounting for primary authorship (*h_p_*) and senior authorship (*h_s_*) are shown. (b) Tornado chart shows percent contribution of *h_p _*and *h_s _*to *h* index for NYC neurosurgeons with different faculty ranks. Increasing academic rank is correlated with increasing percentage of papers with senior authorship.

Advancing academic rank revealed a trend toward an increasing number of *h*_p_ articles. This trend achieved significance when comparing assistant professor to professor (p < 0.0001), associate professor to professor (p = 0.0002), and assistant professor to chair ranks (p = 0.0119). A similar and more robust trend was revealed when examining *h*_s_ articles. This trend achieved significance for all comparisons except assistant professor to associate professor and professor to chair ranks (Figure 2a, Table 5).

 

**Table 5:** Tukey’s multiple comparisons tests for faculty rank at different subindices and percentage subindices (one-way ANOVA). APV: Adjusted p-value; Assoc.: Associate professor; Asst.: Assistant professor; Prof.: Professor; Sig.: Significance.

	h_p_-index		h_s_-index		h_p_/h		h_s_/h			
	Sig.	APV	Sig.	APV	Sig.	APV	Sig.	APV		
Chair vs. Prof.	NS	0.9958	NS	0.5230	NS	0.8414	NS	0.9384		
Chair vs. Assoc.	NS	0.1534	***	0.0003	NS	0.5834	NS	0.8473		
Chair vs. Asst.	*	0.0119	****	<0.0001	NS	0.0962	NS	0.0799		
Prof. vs. Assoc.	***	0.0002	****	<0.0001	NS	0.8430	NS	0.9710		
Prof. vs. Asst.	****	<0.0001	****	<0.0001	*	0.0203	**	0.0025		
Assoc. vs. Asst.	NS	0.2849	NS	0.0909	NS	0.2114	*	0.0166		

Progressive advancement in academic rank also revealed a weak trend from primary authorship toward senior authorship, expressed as a percentage of the *h*-core publications produced for the respective academic ranks. This trend achieved significance for *h*_p_/*h* comparing assistant professor to professor (p = 0.0203) and for *h*_s_/*h* comparing assistant professor to associate professor (p < 0.0166) or assistant professor to professor (p < 0.0025) (Figure 2b, Table 5).

**Figure 3:**
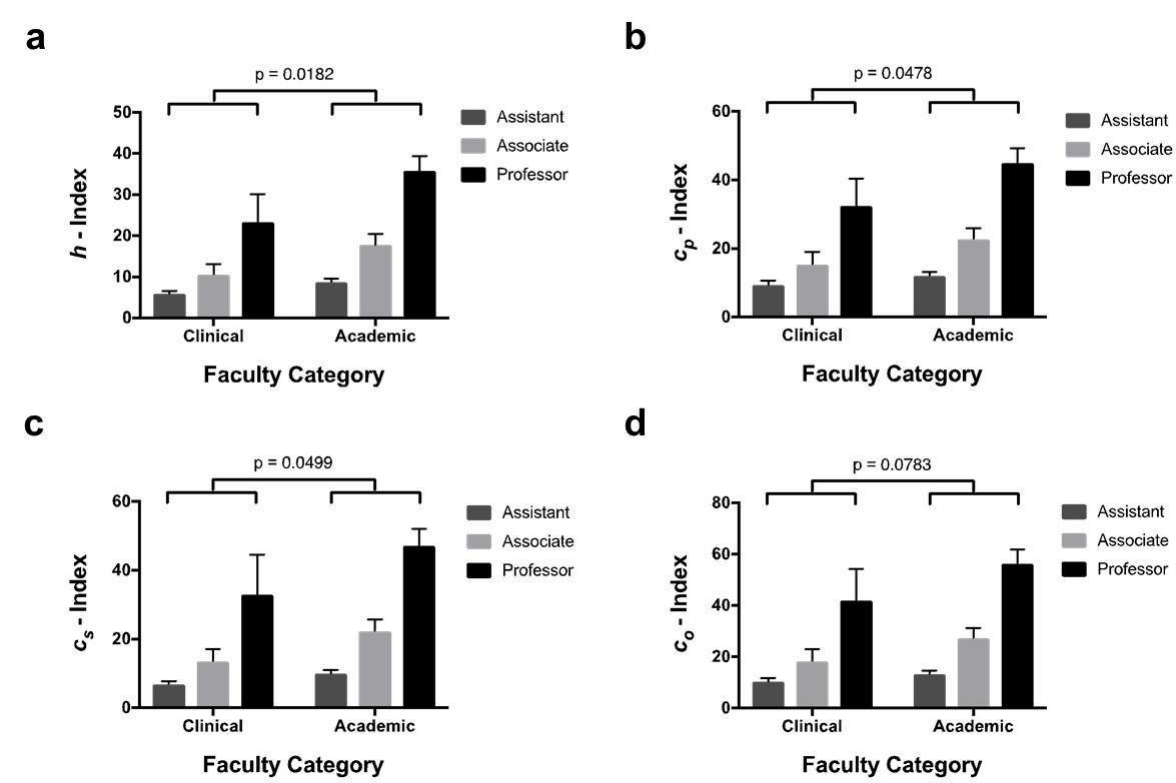
Comparison of academic vs. clinical faculty. Academic *h*, *c*_p_, and *c*_s_ indices are significantly higher than clinical *h*, *c*_p_, and *c*_s_ indices. (a-d) Bar graphs show mean (SEM) index values for academic and clinical New York City (NYC) neurosurgeons with different faculty ranks. p-values show variation between academic and clinical faculty (two-way ANOVA). Graphs a, b, c, and d show mean *h*,* c_p_*,*c_s_*,and*c_o_*indices, respectively.

Faculty members were examined, comparing clinical (i.e., having the words adjunct, affiliate, clinical, courtesy, voluntary, instructor, lecturer, attending, preceptor, or staff in their title) to academic faculty (i.e., assistant professors, associate professors, professors and chairs). Two-way ANOVA was performed and revealed that, compared to clinical faculty, *h*, *c*_p_, and *c*_s_ indices were all significantly higher in academic faculty. This finding did not achieve significance for *c*_o_ (p = 0.0783) and both groups revealed significant differences between *h*-index and academic rank (Figure 3a-3d, Tables 6-7).

**Table 6:** Two-way ANOVA for faculty category at different indices. APV: Adjusted p-value; Sig.: Significance.

	h-index		c_p_-index		c_s_-index		c_0_-index	
	Sig.	APV	Sig.	APV	Sig.	APV	Sig.	APV
Interaction	NS	0.4634	NS	0.5753	NS	0.5855	NS	0.6425
Academic vs. Clinical	*	0.0182	*	0.0478	*	0.0499	NS	0.0783
Faculty category	****	<0.0001	****	<0.0001	****	<0.0001	****	<0.0001

**Table 7:** Tukey’s multiple comparisons tests for faculty rank at different indices (two-way ANOVA). APV: Adjusted p-value; Assoc.: Associate professor; Asst.: Assistant professor; Prof.: Professor; Sig.: Significance.

	h-index		c_p_-index		c_s_-index		c_0_-index	
	Sig.	APV	Sig.	APV	Sig.	APV	Sig.	APV
Prof. vs. Assoc.	****	<0.0001	****	<0.0001	****	<0.0001	****	<0.0001
Prof. vs. Asst.	****	<0.0001	****	<0.0001	****	<0.0001	****	<0.0001
Assoc. vs. Asst.	*	0.0188	*	0.0188	*	0.0197	*	0.0165

**Figure 4:**
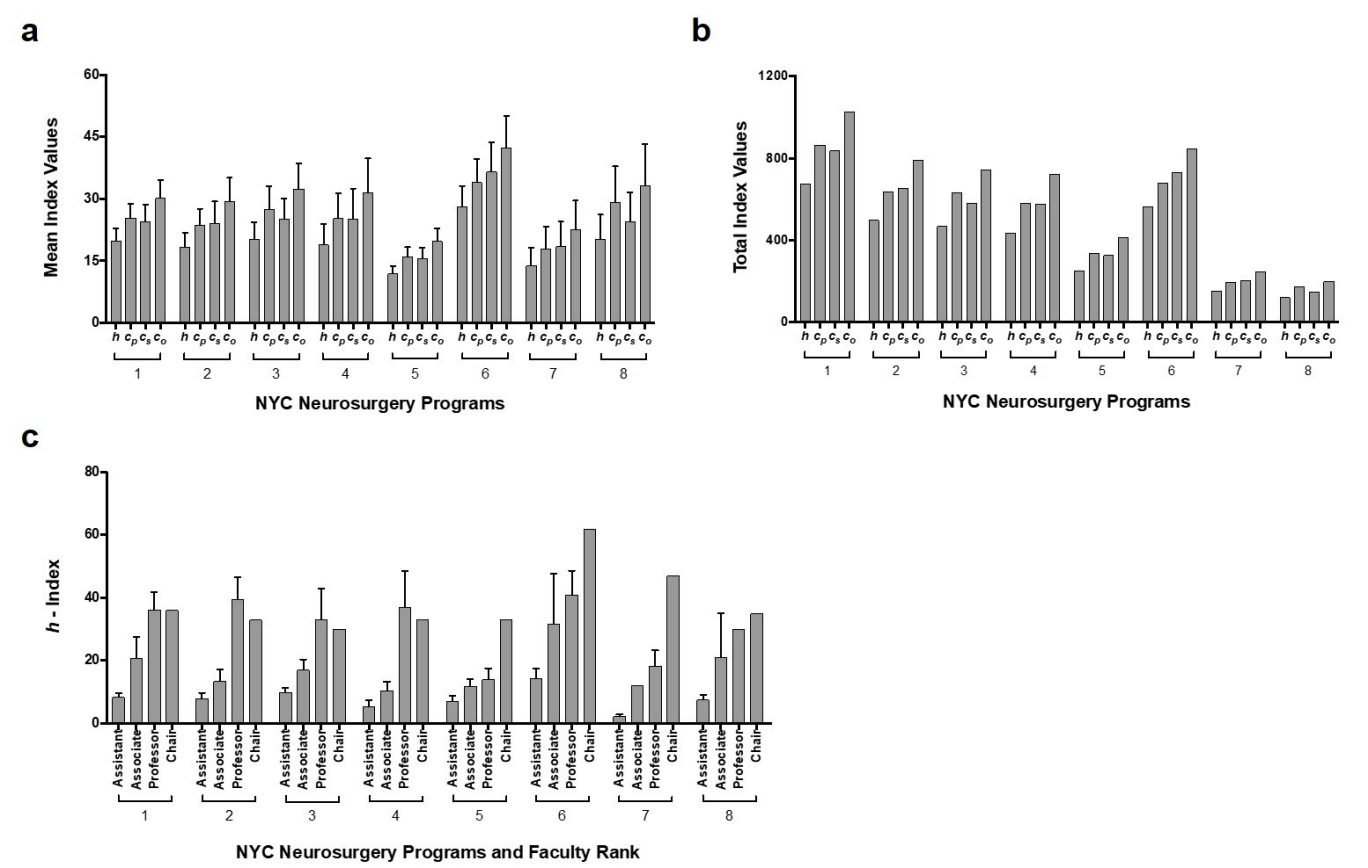
Mean and total h, cp, cs, and co index values for individual New York City (NYC) neurosurgery programs. (a) Mean (SEM) *h*,* c_p_*,*c_s_*,and*c_o_*index values for individual NYC neurosurgery programs in the NYC metropolitan region. (b) Total *h*,* c_p_*,*c_s_*,and*c_o_*index values for programs 1-8. (c) Mean (SEM) *h-*index values for neurosurgeons with different faculty ranks within programs 1-8.

Individual programs were separately evaluated to examine their *h*, *c*_p_, *c*_s_, and *c*_o_ indices (Figure 4a-4b, Table 8). The *c*_p_ for individual programs (1, 3 and 8) was higher than *c*_s_ in programs 1, 3 and 8, while it was lower in programs 2, 6 and 7 or roughly equivalent in programs 4 and 5. A general trend of summed indices increasing with program size was noted, although this was not seen in every case (e.g., Program 6). No significant correlation was revealed between mean *h*, *c*_p_, *c*_s_, and *c*_o_ indices and program size as defined by number of faculty (Table 9). Examination of individual programs showed that mean *h*-index typically increased as faculty rank advanced and that most faculty rank comparisons were not statistically significant. Statistical significance was seen most often comparing assistant professor to professor rank in four of eight programs (Figure 4c, Table 10). This finding, which is inconsistent with Figure 1a, may be due to the limited number of faculty members at each rank in individual programs.

**Table 8:** Overview of indices for individual New York City (NYC) neurosurgery departments.

				h-index		c_p_-index		c_s_-index		c_0_-index	
Program	# Faculty	Min	Max	Mean (SEM)	Total	Mean (SEM)	Total	Mean (SEM)	Total	Mean (SEM)	Total
1	34	2	77	19.88 (3.04)	676	25.46 (3.43)	865.5	24.63 (3.96)	837.5	30.21 (4.35)	1027
2	27	1	72	18.48 (3.35)	499	23.69 (3.92)	639.5	24.22 (5.15)	654	29.43 (5.65)	794.5
3	23	2	94	20.39 (3.98)	469	27.59 (5.49)	634.5	25.28 (4.76)	581.5	32.48 (6.15)	747
4	23	0	113	18.96 (4.94)	436	25.30 (5.95)	582	25.17 (7.30)	579	31.52 (8.23)	725
5	21	3	33	11.95 (1.73)	251	16.14 (2.17)	339	15.62 (2.59)	328	19.81 (2.96)	416
6	20	0	78	28.20 (4.95)	564	34.03 (5.64)	680.5	36.58 (7.05)	731.5	42.40 (7.72)	848
7	11	1	47	13.91 (4.29)	153	17.95 (5.37)	197.5	18.50 (6.04)	203.5	22.55 (7.06)	248
8	6	6	35	20.33 (5.87)	122	29.17 (8.69)	175	24.50 (7.15)	147	33.33 (9.93)	200

**Table 9:** # Faculty vs. mean indices at individual neurosurgery programs.

	Sig.	p-value
# Faculty vs. mean h	NS	0.7952
# Faculty vs. mean c_p_	NS	0.9865
# Faculty vs. mean c_s_	NS	0.7518
# Faculty vs. mean c_o_	NS	0.9110

**Table 10:** Tukey’s multiple comparisons tests for faculty rank at individual New York City (NYC) neurosurgery programs. APV: Adjusted p-value; Assoc.: Associate professor; Asst.: Assistant professor; Prof.: Professor; Sig.: Significance.

	Program 1		Program 2		Program 3		Program 4		Program 5		Program 6		Program 7		Program 8	
	Sig.	APV	Sig.	APV	Sig.	APV	Sig.	APV	Sig.	APV	Sig.	APV	Sig.	APV	Sig.	APV
Chair vs. Prof.	NS	>0.9999	NS	0.9496	NS	0.9981	NS	0.9973	NS	0.0609	NS	0.7188	*	0.0232	NS	0.9929
Chair vs. Assoc.	NS	0.7077	NS	0.4016	NS	0.8981	NS	0.7280	*	0.0224	NS	0.4757	*	0.0137	NS	0.8472
Chair vs. Asst.	NS	0.2041	NS	0.1835	NS	0.6845	NS	0.5730	**	0.0063	NS	0.1052	**	0.0021	NS	0.5291
Prof. vs. Assoc.	NS	0.1070	**	0.0016	NS	0.3740	NS	0.0986	NS	0.9390	NS	0.8624	NS	0.7010	NS	0.9463
Prof. vs. Asst.	****	<0.0001	****	<0.0001	NS	0.0516	*	0.0237	NS	0.3505	NS	0.0614	*	0.0419	NS	0.6389
Assoc. vs. Asst.	NS	0.1908	NS	0.7458	NS	0.8774	NS	0.9638	NS	0.4713	NS	0.4182	NS	0.3834	NS	0.7860

**Figure 5:**
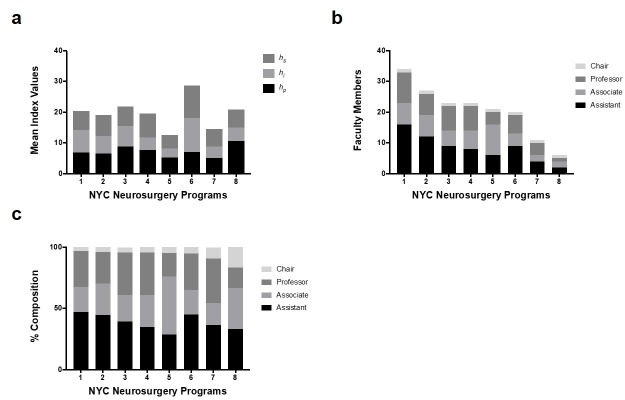
h-core and faculty composition for individual New York City (NYC) neurosurgery programs. (a) Stacked bar graphs show the mean values of *h*_s_, *h*_i_ and *h*_p_ within the eight departments evaluated. (b) Stacked bar graphs show the number of faculty at each rank within the eight departments evaluated. (c) Stacked bar graphs show the relative percentages of faculty at each rank within the eight departments evaluated.

Departments were also evaluated to show the mean values of *h*_p_, *h*_i_, and *h*_s_ for each program (Figure 5a), the absolute composition (Figure 5b) and the percentage composition (Figure 5c) of faculty rank at each institution. No significant correlation was seen between mean *h*-index values and percentage of faculty in each rank (Table 11).

**Table 11:** Departmental mean h-index vs. percentage of each academic rank at that program. Assoc.: Associate professor; Asst.: Assistant professor; Prof.: Professor; Sig.: Significance.

	Sig.	p-value
Mean h vs. % Asst.	NS	0.0931
Mean h vs. % Assoc.	NS	0.1961
Mean h vs. % Prof.	NS	0.7396
Mean h vs. % Chair	NS	0.9302

**Figure 6:**
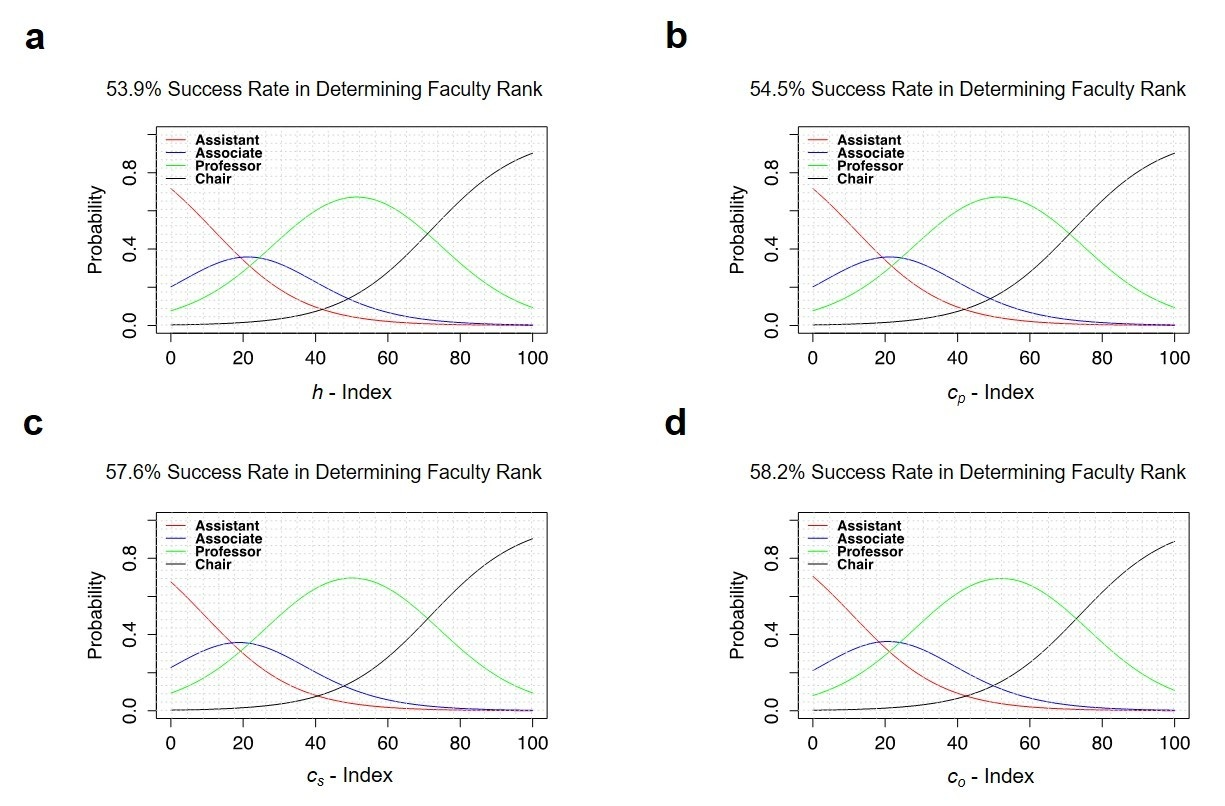
Success rate of different indices at predicting faculty rank. (a-d) Proportional odds models for New York City (NYC) neurosurgeons estimating probability of having a specific faculty rank based on index values. Graphs a, b, c, and d show forecasted probability as a function of *h*,* c_p_*,*c_s_*,and*c_o_*indices, respectively. Although not statistically significant, *c*_o_ index has a higher in-sample success rate than *h*-, *c*_p_- or *c*_s_-indices in predicting faculty rank.

Proportional odds models showed that an author’s *c*_o_-index was more likely to predict faculty rank than their *h*-index, though this did not achieve statistical significance (see Figure 6).

## Discussion

The *h*-index was first used in the neurosurgical literature by Lee et al. [9] in 2009, whose evaluation of 30 neurosurgical programs confirmed a positive correlation between *h*-index and academic rank. Larger studies by Spearman et al. [12] (1120 neurosurgeons in all Electronic Residency Application Service-listed training programs) and Campbell et al. [13] (986 faculty members in 97 academic neurosurgery departments) confirmed the correlation between *h*-index and academic rank. Comparisons have also been made across neurosurgical departments [14], within [15], and across different neurosurgical subspecialties [16].

During the past seven years, a steady increase has been noted in the application of bibliometrics to the field of neurosurgery [17-19], with deeper analysis of randomly selected individuals and training programs [9], academic departments and residency programs [14, 20], resident productivity [21], fellowship training [22], subspecialties of pediatric [15] and spine surgery [23], gender [24] and National Institutes of Health (NIH) funding [25].

Authorship value has been studied previously in attempting to address the reality that different authors collaborating on a single publication contribute to different degrees and in different ways. Bornmann’s study of 37 *h*-index variants noted six that addressed multi-authorship [5]. Batista’s *h*_I_ allowed for comparison between researchers in different fields and suggested the number of papers a researcher would have written had he or she worked alone [26]. Opthof and Wilde proposed examining the *h*-indices of only first-authored papers [27]. Schreiber proposed the *h*_m_-index to fractionalize the counting of papers, taking multi-authorship into account [28] and offered the *h*_ms_ to account for multiple authors and self-citation [29]. Hu et al. [30] proposed *h*_maj_ to denote the *h*-index where the author played “a major or core role” by being a first or corresponding author. Lee et al. [9] randomly selected 30 academic neurosurgical programs and performed a weighted *h*-index calculation on five individuals of each academic rank at each institution “with full credit for first and last authorship, half credit for second authorship, and a quarter credit for any other contributing authorship.” The study found that “the weighted *h*-index did not appear to differ from the non-weighted *h* index.”

Subsequently, Khan et al. [19] studied 40 neurosurgeons randomly selected from 188 neurosurgeons at 11 academic programs. They determined an authorship value (AV) by applying a value of one to the first author, 0.75 to the last author, and 0.25 to any other author position. They found that AV varied with academic rank in a pattern different from that of *h*-index, noting that associate professors had the lowest value and that, overall, the AV of *h*-core articles was lower than that of *h* articles. These findings led the researchers to postulate that associate professors at the midpoint of their careers might produce more collaborative contributions to publications compared to first-authored papers (early in their careers) or senior-authored papers (later in their careers). They acknowledged, however, that their study had yielded no specific data to support that hypothesis.

Consistent with the findings of previous research, this study has confirmed a relationship between *h*-index and academic rank across a subset of academic neurosurgical programs. This finding was statistically significant between all academic ranks except between chair and professors. Some prior studies have shown a positive relationship [9] between the professor and chair rankings. This study adds to the literature, recognizing that the findings derive from the evaluation of a small (n = 8) number of programs.

In our *c*-index calculations, we chose to give enhanced credit to the first and last authors, based on the presumption that they contributed most to the creation of the scientific hypothesis, the research method and analysis of the findings, organization and authorship of the paper, and assumed the greatest responsibility for project oversight. Second and second-to-last authors were presumed to have provided similar contributions but to a lesser degree with less enhanced credit. During calculation of the *c*-indices, *i*_p_ and *i*_s_ represented the increase in *h*-index due to the primary and senior authorship positions, respectively. This method of assigning author position and associated point value is proposed as more granular to allow the generation of the *h*_p_, *h*_i_, and *h*_s_ indices and the subsequent calculation of *c*_p_, *c*_s_ and *c_o_* indices. We present these tools as a framework for calculation, recognizing that future researchers may choose different numbers for their weighting systems.

*c*_s_, *c*_p_ and *c*_o_ are, by definition, additive to an author’s *h*-index and one could assume that they would follow trends similar to those found with an author’s *h*-index. It is interesting to note that *c*_o_ has a greater success rate in predicting faculty rank, though nonsignificant. If a higher success rate, or greater correlation between the c-indices and faculty rank could be confirmed, then one could compare the *c*-index of a faculty member with their current rank to suggest whether they had already achieved enough, academically, to recommend appropriate academic promotion (*c*-index higher than the predicted academic rank) or had achieved their academic rank based on factors other than academic publishing (clinical track, other teaching activities, departmental leadership). The absence of statistical significance of *c*_o_-index as a better predictor of faculty rank may be due to the small sample of eight neurosurgery programs. Nevertheless, the proportional odds model can be an important tool in having a way to quantitatively compare how well various indices can successfully predict faculty rank amongst one another.

Because *h*_p_ represents the number of first- and second-author papers in the author’s *h*-core, it may be of greatest interest when looking at an individual’s ability to develop and execute novel research and/or publications. Likewise, *h*_s_ represents the contribution of senior and second-to-senior-author papers to the author’s *h*-index and may be of greatest interest when evaluating the ability of a senior researcher to mentor multiple people, run multiple laboratory projects, or generate novel research ideas and supervise their execution by junior members of the research team. Finally, *h*_i_ represents the remaining contribution of internal authorship to the author’s overall *h*-index. This format for organizing the *h*-index allows evaluators to determine which aspect of an individual’s academic profile is most relevant to their pursuits. It also allows investigators to self-evaluate and determine what type of responsibilities they wish to undertake on future academic projects.

Finally, we recognize that all these indices are based on citation metrics and that there are researchers with publications that have been cited fewer than *h* times. These may be publications that are very recent, in small academic fields, in journals not typically included in the more common publication databases or that have been cited infrequently. We do not consider these publications to lack merit, only strength of citation. We have proposed the *h*_p_, *h*_i_, and *h*_s_ indices as tools to better represent the data used to create an author’s *h*-index. Accordingly, we also propose a *p*-index to represent all of an author’s publications, including those not yet cited or those cited fewer than *h* times. *p*_p_, *p*_i_, and *p*_s_ will represent primary, internal and senior authorship indices of these publications, respectively.

Study limitations

A limitation of this study is the small number of academic neurosurgical programs analyzed. We selected a small subset of programs to test the initial application of novel metrics. Going forward, we plan to apply these indices to the full complement of academic neurosurgical programs in the United States to better evaluate the merits of these indices. Doing so should reveal whether our findings are confirmed in a larger setting and whether the addition of more chairs, in particular, yields greater statistical significance.

The identification of appropriate faculty members for inclusion is dependent on the publicly available website data. We presume that most departments’ listed information is relatively stable and that changes in personnel and their academic rank are relatively rare.

Every effort was made to ensure that the data used for *h*-index calculations was accurate. In particular, the Scopus database was used because each individual has a unique author profile with respect to identification of the articles they have published. Previously, Scopus did not cite articles prior to 1996, potentially resulting in incomplete data, unless a labor-intensive manual calculation of pre-1996 citations was performed. This has been done in other studies that have postulated that exclusion of pre-1996 citations should have less impact as older researchers (with pre-1996 citations) retire [16]. Since 2014, Elsevier has been adding pre-1996 references to the Scopus library to enable accurate searches back to 1970.

## Conclusions

We have introduced *h*_p_, *h*_i_and* h*_s_ as novel indices to more accurately and consistently describe the authorship makeup of an individual’s *h*-core articles. We have introduced *c*_p_, *c*_s_and* c*_o_ indices as novel, weighted variants of the *h*-index that adjust for increased participation of authorship in enhanced positions. Applying these to a subset of United States academic neurosurgical programs, we have shown increasing numbers of articles with enhanced position authorship and a shift towards increasing percentage of senior authorship as academic rank increases. We believe these tools will provide a more accurate and reliable picture of an author’s publications that should be of use to the academic community.
